# A Fast Synthetic Aperture Radar Raw Data Simulation Using Cloud Computing

**DOI:** 10.3390/s17010113

**Published:** 2017-01-08

**Authors:** Zhixin Li, Dandan Su, Haijiang Zhu, Wei Li, Fan Zhang, Ruirui Li

**Affiliations:** College of Information Science and Technology, Beijing University of Chemical Technology, Beijing 100029, China; lizhixin@bi.a.u-tokyo.ac.jp (Z.L.); 2014210360@grad.buct.edu.cn (D.S.); zhuhj@mail.buct.edu.cn (H.Z.); liw@mail.buct.edu.cn (W.L.)

**Keywords:** cloud computing, synthetic aperture radar (SAR), raw data generation, distributed simulation, big data

## Abstract

Synthetic Aperture Radar (SAR) raw data simulation is a fundamental problem in radar system design and imaging algorithm research. The growth of surveying swath and resolution results in a significant increase in data volume and simulation period, which can be considered to be a comprehensive data intensive and computing intensive issue. Although several high performance computing (HPC) methods have demonstrated their potential for accelerating simulation, the input/output (I/O) bottleneck of huge raw data has not been eased. In this paper, we propose a cloud computing based SAR raw data simulation algorithm, which employs the MapReduce model to accelerate the raw data computing and the Hadoop distributed file system (HDFS) for fast I/O access. The MapReduce model is designed for the irregular parallel accumulation of raw data simulation, which greatly reduces the parallel efficiency of graphics processing unit (GPU) based simulation methods. In addition, three kinds of optimization strategies are put forward from the aspects of programming model, HDFS configuration and scheduling. The experimental results show that the cloud computing based algorithm achieves 4× speedup over the baseline serial approach in an 8-node cloud environment, and each optimization strategy can improve about 20%. This work proves that the proposed cloud algorithm is capable of solving the computing intensive and data intensive issues in SAR raw data simulation, and is easily extended to large scale computing to achieve higher acceleration.

## 1. Introduction

Due to the imaging characteristics of high resolution, day-and-night and weather-independent, Synthetic Aperture Radar (SAR) has been widely used for Earth remote sensing for more than 30 years, and it has come to play a significant role in geographical surveys, climate change research, environment and Earth system monitoring, multi-dimensional mapping and other applications [1]. In the foreseeable future, more multi-platform, multi-mode and multi-band SAR systems will be developed to satisfy the practical demands. Due to the time consuming and high-cost of SAR flight experiments, computer simulation is often applied to assist the key technology research, system design, system development, and even the data applications. In order to fulfill aforementioned support, the accurate and reliable raw data that contain various actual system errors and simulate large areas are necessary. Thus, the requirement poses a challenge for SAR raw data simulation accuracy and efficiency.

Currently, the SAR raw data simulation algorithm can be mainly divided into two categories: forward processing and inverse processing. The forward processing algorithms simulate the physical process of microwave transmitting and receiving, and then calculate the SAR raw data, including the time domain pulse coherent algorithm [2], the frequency domain pulse coherent algorithm [2], the 2D frequency domain algorithm [3] and its improved algorithms [4,5]. Comparatively, the inverse processing algorithms simulate the SAR raw data through the inverse SAR imaging processing, including the inverse fourth-order extended exact transfer function (EETF4) algorithm [6], the inverse ω−κ algorithm [7,8], the inverse Chirp Scaling algorithm [9] and the inverse frequency scaling algorithm [10]. Furthermore, the 2D frequency domain algorithm and the inverse processing algorithm are efficient although difficult for considering the actual system errors. To account for these errors, some assumptions need to be introduced into these algorithms, resulting in the loss of accuracy and efficiency to some extent. On the other hand, the time-domain algorithm can easily consider the systematic errors and motion errors, so it is always employed for practical SAR simulators [11,12].

With the increased spatial resolution and swath width of SAR systems, the simulated targets increase massively, which causes the rapid growth of computational time. Although the time-domain SAR raw data simulation algorithm has been improved for smaller time complexity, the optimization still does not achieve satisfactory performance. Due to the independence of raw signal collection, parallelization is the most straightforward idea and can greatly shorten the simulation time with state-of-the-art high performance computing (HPC) technologies. Thus, several classical HPC methods have been introduced into the SAR raw data simulation for speedup, such as open multiple processing (OpenMP) with multi-cores [13], message passing interface (MPI) with multi-CPUs [14], grid computing with multi-computers [15] and graphics processing unit (GPU) computing with massive cores [16]. These methods are all computing oriented, and seldom consider the big data input/output (I/O) solution. On the other hand, the cost of these methods are high in that the required computers and servers are expensive and energy consuming. Furthermore, the last decades have seen an unprecedented development in the remote sensing industry, which demands a big data solution of data producing and applications. Compared with these HPC technologies, cloud computing is the best solution for these three issues.

Cloud computing is a large-scale distributed computing paradigm that is driven by economies of scale, in which a pool of abstracted, virtualized, dynamically-scalable, managed computing power, storage, platforms and services are delivered on demand to external customers over the internet [17]. To realize these merits, programming model, distributed storage, data management and virtualization constitute the key technologies of cloud computing. Programming model is mainly used to solve the large-scale distributed computing issue. The most popular programming model is Google’s MapReduce [18], which simplifies the distributed programming process only through map and reduce function design. Then, the cloud system will automatically manage the specific task partition, scheduling, processing and storage. In addition, there are some other similar programming models, such as Dryad [19], Pregel [20] and so on. Distributed storage technology is a key to the solution of the data intensive issue. It spreads the single node pressure of data access to multiple nodes, thereby breaking the data access bottleneck. The frequently-used distributed data storage systems are, respectively, Google File System (GFS) [21] and Apache Hadoop Distributed File System (HDFS) [22]. Based on distributed storage technology, the distributed data management system is employed to handle the big data issue over the Petabyte level, e.g., Google BigTable [23] and Amazon Dynamo [24]. Virtualization is the key technology to integrate various computing and storage resources, and makes them available for different levels of users. With the development of cloud computing technologies, several cloud computing platforms have sprung up to support the big data applications, such as IBM’s Blue cloud, Google cloud, Amazon elastic cloud, Apache Hadoop and so on. With the further application of cloud computing, some new programming models have emerged to enhance the computing ability over the MapReduce model, including Tez [25], Spark [26] and Storm [27]. Meanwhile, a new resource management framework, named YARN [28] has been introduced into cloud computing to separate the resource management and task scheduling, and has brought great benefits for resource utilization and data sharing.

As the outstanding capacity of the cloud computing framework, the MapReduce implementations of classical algorithms have drawn increasing attention. Cloud computing has been applied to remote sensing processing [29], geoscience [30], SAR interferometry [31], image processing [22] and other remote sensing areas. Cloud computing is the future trend of the remote sensing big data processing. Predictably, cloud computing not only boosts the big data I/O efficiency, but also improves the processing efficiency by large scale computing resources. Therefore, cloud computing is first introduced to the SAR raw data simulation for an initial attempt of service-oriented solutions. Compared to previous work [11,15,16,32], we make the following contributions:

- a first cloud computing implementation for SAR raw data simulation;
- applying the MapReduce model for irregular accumulation of SAR return signals, which is a hard issue for fine-grained parallelization, like GPU;
- optimizing the computing efficiency through introducing combine method, tuning of Hadoop configuration and scheduling strategies.

The rest of the paper is organized as follows: Section 2 briefly introduces the SAR raw data simulation algorithm, its parallelization analysis and the principle of cloud computing; Section 3 presents the proposed cloud computing based raw data simulation algorithm. Then, the experimental results and analysis are discussed in Section 4. Finally, conclusions are drawn in Section 5.

## 2. Related Work

In this section, we will briefly introduce some background knowledge on Fast Fourier Transform (FFT) based time domain stripmap SAR raw data simulation and its parallel analysis, cloud computing, respectively. It is noted that the geometry calculation is not discussed in the paper. Except for the classical stripmap mode, other main stream SAR modes, namely the spotlight, sliding spotlight, ScanSAR and Terrain Observation by Progressive Scans (TOPS) SAR modes, perform complex beam steering in azimuth and range direction, and lead to different geometry calculation in raw data simulation. Except for the FFT based time domain raw data simulation of different SAR modes, the kernel part of signal simulations are all the same. Therefore, the proposed cloud computing method can be applied to all of the SAR modes by introducing corresponding geometry calculation steps.

### 2.1. SAR Raw Data Simulation Algorithm

The echo signal model in [33] is applicable for airborne, spaceborne SAR data. Assuming that the transmitting pulse is a linear frequency modulated (FM) signal pulse, i.e.,

(1) $$s_{t}\left(\tau \right)=s_{r}\left(\tau \right)\exp\left(jw_{c}\tau \right)=rect\left(\frac{\tau }{T_{p}}\right)\exp\left(jw_{c}\tau +j\pi k_{r}\tau^{2}\right).$$

Through coherent receiving, the single point echo is expressed as 2D s(t,τ)

(2) $$\begin{array}{c} s\left(t,\tau \right)=\sigma W_{a}\left(\theta \right)rect\left(\frac{t}{T_{a}}\right)\exp\left(-j\frac{4\pi r(t)}{\lambda }\right)\\ \;\;\;\times rect\left(\frac{\tau -\frac{2r(t)}{c}}{T_{p}}\right)\exp\left(j\pi k_{r}{\left(\tau -\frac{2r(t)}{c}\right)}^{2}\right), \end{array}$$

where *t* is the azimuth time, *τ* is the range time, *σ* is the scattering coefficient, $W_{a}$ is the antenna gain, *θ* is the antenna look angle, $T_{p}$ is the signal pulse width, $T_{a}$ is the synthetic aperture time, r(t) is the distance between target point and the radar antenna phase center at time *t*, $k_{r}$ is the signal modulation frequency rate, and rect(·) is a rectangular envelope.

When the simulation objects are distributed targets, the SAR echo signal can be obtained by

(3) $$\begin{array}{c} s\left(t,\tau \right)=\sum_{n=1}^{T}\sum_{i=1}^{M}\sigma_{i}W_{a}\left(\theta_{i}\right)s_{r}\left(\tau -\frac{2r_{i}\left(t_{n}\right)}{c}\right)\\ \;\times rect\left(\frac{t_{n}}{T_{a}}\right)\exp\left(-j\frac{4\pi r_{i}\left(t_{n}\right)}{\lambda }\right) \end{array},$$

where *i* is the order number of distributed points in scattering matrix, *n* is the order number in azimuth time, *T* is the number of azimuth samples, and *M* is the total number of target points.

**Figure 1:**
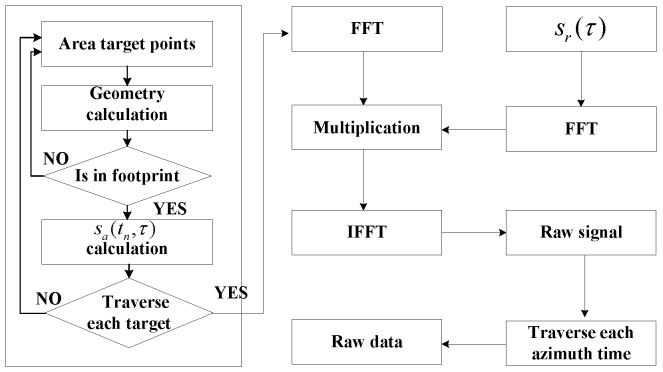
The Fast Fourier Transform (FFT) based time-domain SAR raw data simulation diagram.

In a practical engineering calculation, the FFT based time-domain method, which calculates the scattering target points accumulation by frequency domain multiplication, is often applied for raw data generation as follows:

(4) $$\begin{array}{c} s\left(t,\tau \right)=\sum_{n=1}^{T}s_{a}\left(t_{n},\tau \right)⊗s_{r}\left(\tau \right)\\ \;\;\;=\sum_{n=1}^{T}f^{-1}\{f[s_{a}\left(t_{n},\tau \right)]S_{r}\left(\xi \right)\}, \end{array}$$

with

(5) $$\begin{array}{c} s_{a}\left(t_{n},\tau \right)=\sum_{i=1}^{M}\sigma_{i}W_{a}\left(\theta_{i}\right)\exp\left(-j\left(\frac{4\pi r_{i}\left(t_{n}\right)}{\lambda }\right)\right)\\ \times \delta (\tau -\frac{2r_{i}\left(t_{n}\right)}{c}), \end{array}$$

where f(·) is the Fourier transform operator, $f^{-1}\left(\cdot \right)$ is the inverse Fourier transform operator, and $S_{r}\left(\xi \right)$ is the linear FM Signal spectrum. In the procedure of simulation, the linear FM signal spectrum $S_{r}\left(\xi \right)$ does not change, while the azimuth signal spectrum changes with different scattering points and azimuth time. According to Figure 1 and Algorithm 1, the raw data simulation algorithm includes the following five steps:

1. the linear FM signal spectrum $S_{r}\left(\xi \right)$ is calculated;
2. the azimuth signals of all scattering points are calculated and accumulated into $s_{a}\left(t_{n},\tau \right)$, and then transformed into the frequency domain;
3. the spectrum multiplication of azimuth signal and linear FM signal is completed;
4. the raw signal is achieved by the inverse Fourier transform of results in Step 3;
5. for all the azimuth sampling time, steps are repeated to get the complete simulated raw data.

### 2.2. Parallelization of Raw Data Simulation

According to the stop-and-go model, SAR raw data simulation is a serial time process, and then the coupling of transmitting and receiving pulses at different azimuth time is small. Therefore, we can take the pulses transmitting and receiving as the task unit, which will be dispatched to every computation node and calculated quickly by MPI, grid computing or other parallel technologies.

**Algorithm 1** Serial SAR raw data simulation algorithm.**Input:** The simulated target scattering coefficients $\sigma_{i}$ with size *M*, radar signal spectrum $S_{r}\left(\xi \right)$ with size $N_{r}$ **Output:** SAR raw data: 2D complex array s[n][m] with size $N_{a}\times N_{r}$
 1:**for** each $n\in [0,N_{a}]$ **do** 2: **for** each i∈[0,M] **do** 3:  r[n][i]← compute the distance between radar and target *i*; 4:  θ[n][i]← compute the angle of r[n][i] deviating from the beam center; 5:  **if** θ[n][i]>beamwidth **then** 6:   break; 7:  **end** **if** 8:  $N_{ga}\leftarrow$ compute the range gate number of return signal; 9:  phase[n][i]=−4π·r[n][i]/λ;10:  $s_{a}\left[N_{ga}\right].re+=\sigma_{i}\cdot cos\left(phase\left[n\right]\left[i\right]\right)$;11:  $s_{a}\left[N_{ga}\right].im+=\sigma_{i}\cdot sin\left(phase\left[n\right]\left[i\right]\right)$;12: **end** **for**13: $S_{a}\left(\xi \right)\leftarrow$ compute the FFT of $s_{a}$;14: Multiplication of $S_{a}\left(\xi \right)$ and $S_{r}\left(\xi \right)$;15: $s_{a}\leftarrow$ compute the inverse FFT of the product;16: **for** each $m\in [0,N_{r}]$ **do**17:  $s\left[n\right]\left[m\right]=s_{a}\left[m\right]$;18: **end** **for**19:**end for**


The parallelism of SAR raw data simulation can be divided into a coarse-grained strategy and a fine-grained one, as shown in Figure 1. The traditional parallel approach belongs to the former, which takes the repetitious transmitting and receiving pulses process as one task. The process completes the task assignment through dispatching a reasonable number of simulated pulses to different nodes, CPUs, and CPU cores, as shown in Equation (6), i.e.,

(6) $$s\left(t,\tau \right)=\sum_{k=1}^{m}D{}_{k}^{\prime }=\sum_{k=1}^{m}\sum_{n=T_{k}}^{T_{k+1}}s(t_{n},\tau ),$$

in which $D_{k}^{\prime }$ represents the calculation task of node *k*, and *m* indicates the number of sub-tasks.

Comparatively, the parallel simulation based on GPU is a fine-grained parallel method, which optimizes the largest time-consuming step. The task of every thread is the azimuth signal calculation of a single scattering point and a single sampling point multiplication, as shown in Figure 1 and Equation (7), i.e.,

(7) $$\begin{array}{c} s\left(t,\tau \right)=\sum_{n=1}^{T}f^{-1}\{f[\sum_{i=1}^{M}D^{\prime \prime }{}_{\left(n,i\right)}]S\left(\xi \right)\}\\ =\sum_{n=1}^{T}f^{-1}\{\sum_{j=1}^{N}D^{\prime \prime \prime }{}_{\left(n,j\right)}\}, \end{array}$$

where $D_{\left(n,i\right)}^{\prime \prime }$ is the azimuth signal of point *i* in time $t_{n}$, $D_{\left(n,j\right)}^{\prime \prime \prime }$ is the spectrum product of linear FM signal and azimuth signal at range gate *j* in time $t_{n}$, and *N* is the number of range gates. With parallel task decomposition from coarse-grained $D_{k}^{\prime }$ to fine-grained $D_{\left(n,i\right)}^{\prime \prime }$ and $D_{\left(n,j\right)}^{\prime \prime \prime }$, higher efficiency of the parallel simulation is achieved.

### 2.3. Cloud Computing

The popular cloud computing platform is Hadoop, which was originated from a Google cluster system. It is composed of the common module, the HDFS module, the YARN module and the MapReduce module. Among them, common module is a set of utilities that supports other Hadoop modules, HDFS is a distributed file system that provides high-throughput access to application data, YARN is a framework for job scheduling and cluster resource management and MapReduce is a YARN-based system for distributed processing of big data. For a cloud computing application, MapReduce and HDFS are the core factors of cloud algorithm design.

**Figure 2:**
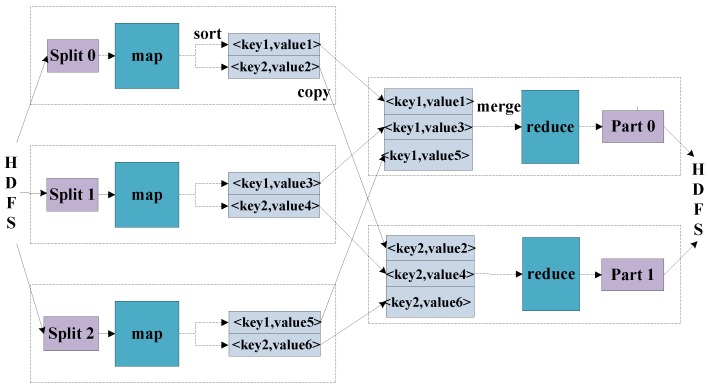
MapReduce model structure.

MapReduce is a programming model for the parallel processing of distributed large-scale data [18]. The whole implementation of MapReduce is mainly divided into two stages: the map stage and the reduce stage, respectively. The inputs and outputs of them are all based on the form of <key,value> pairs, whose data types can be conveniently modified by the programmer. In some cases, more than one value needs to be output, and the interface should be modified in addition to the SAR raw data simulation. Due to raw data being complex data, a complex type is assigned for the pair as <key,(value.re,value.im)>. Firstly, the MapReduce process divides all the data sources into pieces, and dispatches them to the map tasks for them to deal with. Then, the intermediate <key,value> pairs produced by the Map function are buffered in memory. Secondly, the reduce tasks merge all the intermediate values that are associated with the same key, as shown in Figure 2.

**Figure 3:**
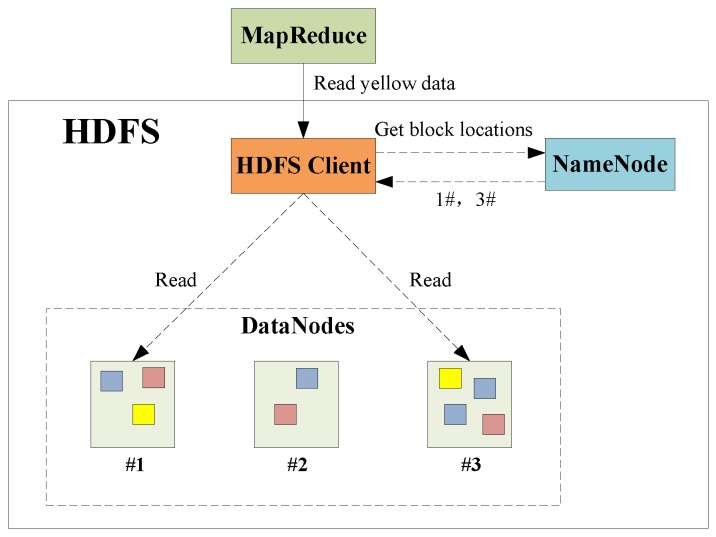
Hadoop Distributed File System (HDFS) structure and its reading process.

The HDFS is a master–slave structure system, as shown in Figure 3. It consists of client, namenode and datanode, which are, respectively, responsible for the execution of the internal and external instructions, the management of the file system name space and the management of cluster data storage. Taking data reading for example, the MapReduce firstly requests the HDFS client to read the yellow type data. Secondly, the HDFS client queries the namenode for detail data block information. Then, the HDFS client contacts the responding DataNodes directly and requests the transfer of the desired data block. Otherwise, data stored in HDFS can be divided into multiple independent data blocks. A read/write operation in HDFS is composed of multiple datanodes’ simultaneous read/write operations, thus boosting the I/O operation efficiency.

## 3. Cloud Implementation of Raw Data Simulation

### 3.1. Cloud Framework

**Figure 4:**
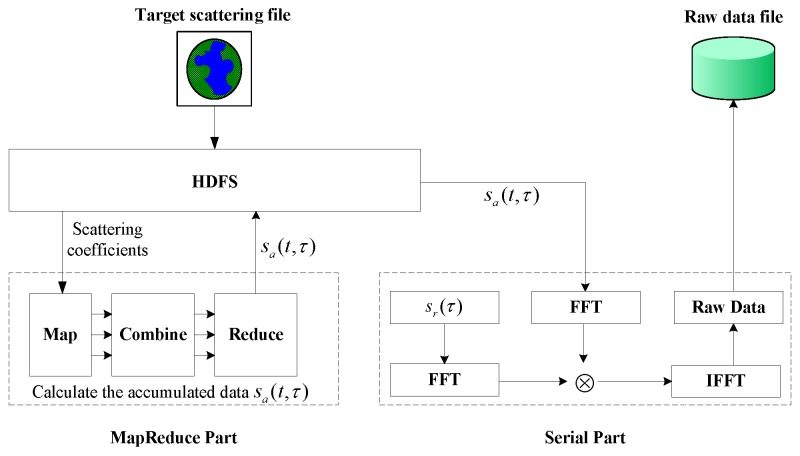
The cloud computing based SAR raw data simulation diagram.

Compared with current super computing technologies, cloud computing is low-cost, large scale and more suitable for industrialized application. In order to make the remote sensing from scientific research to industry, and bring more extensive applications, we propose a cloud computing based SAR raw data simulation to implement a preliminary attempt. What we have designed is a kind of hybrid computing mode. According to Equation (7) and Figure 1, the accumulation of $D_{\left(n,i\right)}^{\prime \prime }$, namely $s_{a}\left(t,\tau \right)$, is the most time-consuming calculation, and is several orders of magnitude higher than other calculations, like FFT, multiplication and inverse Fast Fourier Transform (IFFT). For the minor calculation, the overhead of MapReduce execution is even higher than serial execution. Hence, the accumulation of $s_{a}\left(t,\tau \right)$ is designed with a MapReduce model, and the other modules are processed in serial mode, as shown in Figure 4.

The detailed algorithm is described in Algorithm 2. Firstly, the simulated target scattering file is input into the HDFS. Secondly, the MapReduce model calculates the accumulated data $s_{a}\left(t,\tau \right)$ according to the inherent simulation parameters and the target scattering coefficients from HDFS. Thirdly, the serial program reads the $s_{a}\left(t,\tau \right)$ from HDFS, completes the rest simulation steps, and gets the whole SAR raw data file in the end. Based on the hybrid method, Equation (7) is modified as follows:

(8) $$\begin{array}{c} s\left(t,\tau \right)=f^{-1}\{f[\sum_{n=0}^{T}\sum_{i=1}^{M}D^{\prime \prime }{}_{\left(n,i\right)}]S\left(\xi \right)\} \end{array}.$$

**Algorithm 2** Cloud computing based raw data simulation algorithm.**Input:** The simulated target scattering coefficients $\sigma_{i}$ with size *M*, radar signal spectrum $S_{r}\left(\xi \right)$ with size $N_{r}$ **Output:** SAR raw data: 2D complex array s[n][m] with size $N_{a}\times N_{r}$
1: 2:/*MapReduceprocess*/3:{4:$s_{a}\leftarrow$ the SAR accumulated data in all azimuth time with size $N_{a}\times N_{r}$ ;5:}6: 7:**for** each $n\in [0,N_{a}]$ **do**8: $S_{a}\left[n\right]\leftarrow$ compute the FFT of $s_{a}\left[n\right]\in \left(s_{a}\left[n*Nr\right],s_{a}\left[n*Nr+Nr-1\right]\right)$;9: Multiplication of $S_{a}\left[n\right]$ and $S_{r}\left(\xi \right)$;10: $s_{a}\left[n\right]\leftarrow$ compute the inverse FFT of the product;11:**end for**12: 13:**for** each $n\in [0,N_{a}]$ **do**14: **for** each $m\in [0,N_{r}]$ **do**15:  $s\left[n\right]\left[m\right]=s_{a}\left[n*N_{r}+m\right]$;16: **end** **for**17:**end for**


### 3.2. MapReduce Model of Coherent Accumulation

The accumulation of the azimuth signal $s_{a}\left(t,\tau \right)$ is not only a coherent accumulation in radar principle, but also an irregular accumulation in parallel computing. From Algorithm 1, it can be seen that each return signal should be accumulated in its responding range gate unit $s_{a}\left[N_{ga}\right]$ to meet the requirement of coherent accumulation. Meanwhile, lots of return signals come from discontinuous distributed targets, which are also dynamically changed with the movement of radar footprint. For such an irregular accumulation, the number and location of accumulations are unpredictable. It actually brings difficulties and challenges to parallel computing, such as GPU parallel. In the case of GPU implementation, the irregular accumulation requires the participation of all threads, yielding the access conflicts. The access conflicts of parallel computing will lead to miscalculation or an incorrect cumulative result. To avoid such problems, the method of thread synchronization lock has been considered, such as atomic operation. The essence of atomic operation is to ensure the single thread access to resources, while leaving the other threads in a waiting state. Therefore, the parallel computing efficiency is reduced several times.

For the irregular accumulation, MapReduce is a good solution through the multi-node distributed reduction, which is realized by a multi-level system of map-combine-reduce. The map module is employed for the calculation of accumulation location key and signal value, which are used to construct the $<key,s_{a}\left[key\right]>$ key pairs. The combine module is introduced to carry out the preliminary accumulation among the same range gate, consequently decreasing the overhead of data transmission. Finally, the reduce module merges all the data blocks to finish the MapReduce process, as shown in Algorithm 3. In general, the irregular accumulation is efficiently solved through parallel computing by the map modules, and multi-level reduction by the combine and reduce modules. Compared with the parallel calculation and serial accumulation of the GPU method, the MapReduce process is totally distributed parallel. In the sense, cloud computing outperforms the data intensive oriented GPU method.

**Algorithm 3** Cloud computing based raw data simulation: MapReduce part.**Input:** The simulated target scattering coefficients block $\sigma_{p}$ with size M/l, where *l* is the total number of MapReduce tasks and *p* is the index of simulated target scattering coefficient. **Output:** SAR accumulated data with partial energy: 1D complex array $s_{a}^{\prime }$ with size $N_{a}\times N_{r}$
1: 2:$\mathrm{map}<p,\sigma_{p}>$3:**for** each $n\in [0,N_{a}]$ **do**4: r← compute the distance between radar and target *p*;5: θ← compute the angle of *r* deviating from the beam center;6: **if** θ>beamwidth **then**7:  continue;8: **end** **if**9: $N_{ga}\leftarrow$ compute the range gate number of return signal;10: $key=n\times N_{r}+N_{ga}$;11: phase=−4π·r/λ;12: $s_{a}\left[key\right].re=\sigma_{p}\cdot cos\left(phase\right)$;13: $s_{a}\left[key\right].im=\sigma_{p}\cdot sin\left(phase\right)$;14: $output<key,s_{a}\left[key\right])>$15:**end for**16: 17:combine<key,val>18:**for** each i∈val list **do**19: Tmp.re+=val[i].re;20: Tmp.im+=val[i].im;21:**end for**22:output<key,Tmp>23: 24:reduce<key,val>25:**for** each i∈val list **do**26: Tmp.re+=val[i].re;27: Tmp.im+=val[i].im;28:**end for**29:output<key,Tmp> /*$s_{a}^{\prime }\left[key\right]$=Tmp*/


## 4. Experimental Results and Analysis

### 4.1. Experiment Setup

In this section, we design three experiments to discuss the efficiency of the proposed cloud computing method, and analyze the optimization of the MapReduce model, HDFS configuration and scheduling strategies. The experiments are performed in a cloud environment with eight nodes, and the specific software and hardware information is shown in Table 1.

**Figure 5:**
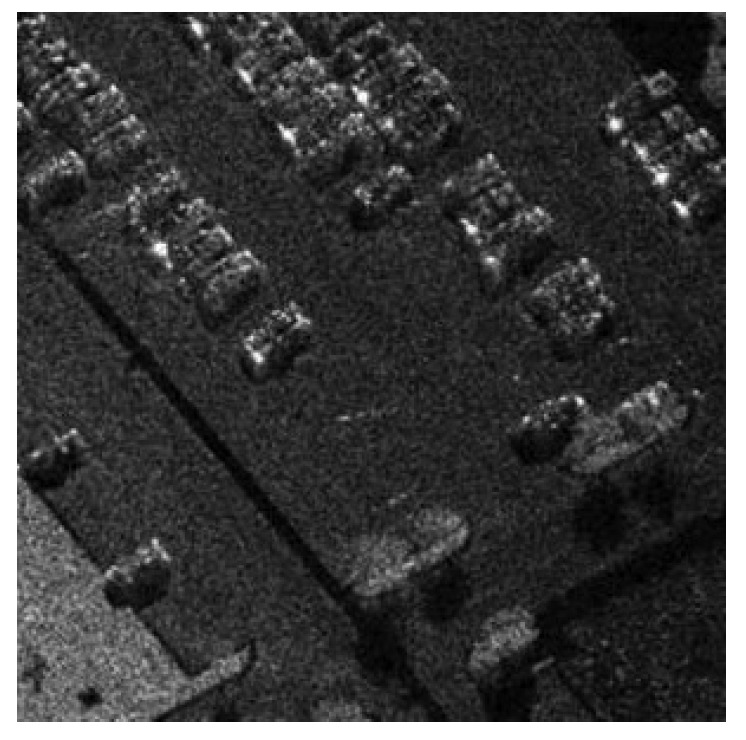
The input target scattering file.

To analyze the performance of cloud computing, we compare three approaches, namely Alg1 with the serial algorithm, Alg2 with the proposed cloud algorithm and Alg3 with the cloud algorithm considering map and reduce only, as shown in Table 2. The difference between Alg2 and Alg3 is whether the combine module does the accumulation tasks. Through the comparison, a conclusion of cloud performance and MapReduce strategy can be drawn. Otherwise, a local SAR image [34] with size 300×300 is taken as the input target scattering file, as shown in Figure 5. The size of simulated area is set to be 4096×4096, which can guarantee a certain amount of computing and data volume for 8-node cloud computing.

### 4.2. Accuracy Analysis

**Figure 6:**
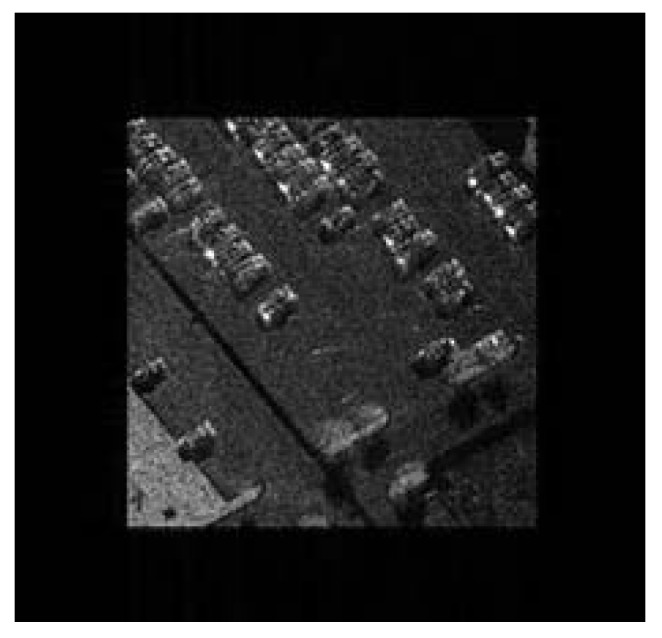
The imaging results of simulated SAR raw data.

Basically, there is no accuracy difference between the serial version and cloud version, in that the program language, data type and simulation principles are all the same. The execution mode of serial or cloud only determines the computing efficiency, rather than the accuracy. To verify the accuracy of cloud algorithm, the numerical comparisons of raw data and imaging results are performed and prove that the results are identical. The imaging results of SAR raw data is shown in Figure 6. Due to the simulated area being bigger than the target area, the image data are zeros outside the target area.

### 4.3. Performance Analysis

Firstly, we conduct an experiment to discuss the performance and optimization of the cloud algorithm. From Table 3 and Table 4, it can be seen that the run time of cloud algorithms are reduced with the increase of node number, and their speedups gradually become worse. Even so, the results are reasonable in that, with the increase of node number, the extra overhead of communication and data transmission is greater than the computing cost savings. In literature [31], the speedup of differential SAR interferometry processing is less than 8× under the cloud environment of 16 nodes. It can be seen that the cloud computing method intends to solve the scheduling issue and distributed data input/output (I/O) issue, which are the lacks of other parallel computing methods, such as GPUs with massive cores [16], open multiple processing (OpenMP) with multi-cores [13], and distributed computing (DC) with multi-CPUs [15]. The parallel computing of cloud, multi-cores and multi-CPUs are based on the CPU platform, which is designed for the control and logic processing. The accelerating effects of these methods are proportional to the number of CPUs. In order to compare these parallel methods, we have carried out another experiment with big input scattering file size of 1000×1000, which is the same simulation condition as the distributed computing method [15]. The employed big input scattering file is achieved by interpolation of Figure 5. The experiment results demonstrate that the cloud computing is faster than the traditional DC. Compared to CPU parallel computing, GPU parallel computing achieves acceleration of a dozen to several hundred times [16]. In terms of computing efficiency, GPU methods are superior to the CPU based methods, namely cloud computing and other CPU parallel methods, which are basically the same efficiency level. Despite this, cloud computing outperforms GPUs and other CPU parallel methods in the distributed network and tremendous scalability. For better use of the cloud algorithm, the data scale and network performance should be further improved. Therefore, we plan to conduct some further research about integrating the cloud computing with GPU in the future.

**Figure 7:**
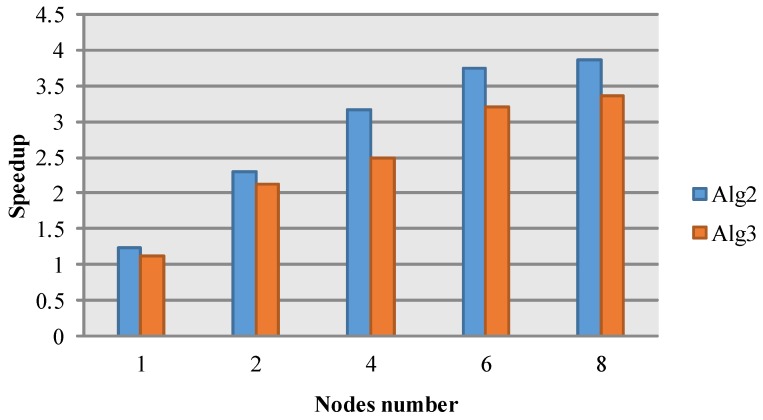
The speedup comparison between Alg2 and Alg3.

The comparison of speedup and parallel efficiency are shown in Figure 7 and Table 5. It can be seen that the Alg2 outperforms Alg3 in the two indicators. The introducing of combine module shows a better speedup, as we expect. Theoretically, the combine module merges the <key,value> pairs with same key in one node, and greatly reduces the data volume that was sent to the reduce module, and then accelerates the cloud computing. For the data intensive issue, the preliminary data merger by the combine module can save the data transfer time and improve the overall simulation efficiency. Although the parallel efficiency reduces with the number of nodes, the computing time is correspondingly decreased. As for cloud computing, it is a common issue that the parallel efficiency is reduced with the decrease of overall computing time. Specifically, there are two reasons for the parallel efficiency’s drop in the experiment. First, the input file data is too small to reflect the advantage of distributed file processing. Second, the cost of nodes scheduling, data exchange and consolidation will increase with the number of nodes. Compared with the employed 100 MB network in our experiments, this issue also exists in the cloud platform with an InfiniBand network, e.g., the parallel efficiency reduces from 57% to 36% when the node number increases from eight to 16 [35].

**Figure 8:**
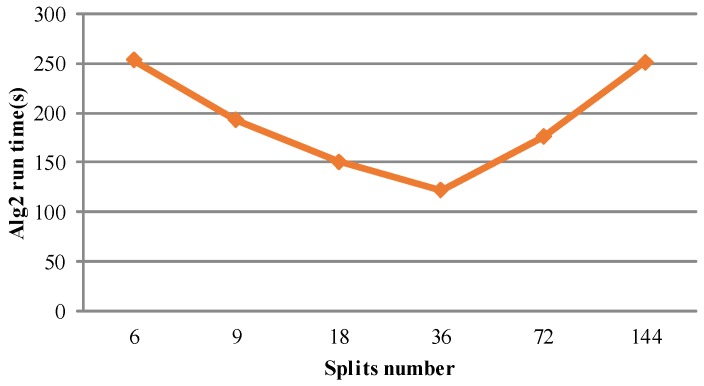
The relationship between split number and simulation time.

Secondly, we mainly discuss the impact of the number of data split on cloud algorithm performance. The number of data split is related to the HDFS configuration. By default, the size of data split is 64 MB. In the MapReduce model, one split inputs into one map module. Thus, the number of data split is the same as the number of map task. For a large scale cloud platform, a vast number of map tasks will lead to excessive data transmission and task startup overhead, and fewer numbers of map tasks will induce lower machine utilization. Therefore, we conduct another experiment under the condition of four nodes, Alg2, and different split number. The simulation results are shown in Table 6 and Figure 8 and prove the aforementioned analysis. In the experiment, each node has eight containers for map tasks. Therefore, the total number of containers is 32. When the map task number is six, all of the tasks run on one node. However, for 36 map tasks, all four of the nodes are busy computing. In addition, although all the nodes are busy with 144 map tasks, the experiment spends more time in that there are more task startup and data transmission overhead consumed. To sum up, it explains why the algorithm is most efficient with 36 data splits.

**Figure 9:**
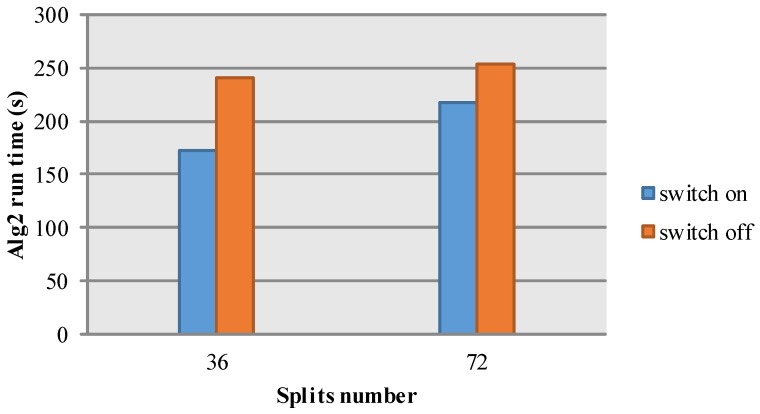
The FFT based time-domain SAR raw data simulation diagram.

Thirdly, we mainly discuss the impact of scheduling strategy. Speculative task is an important optimization strategy in the MapReduce model, and is especially suitable for the cloud environment of unbalanced loading and poor networks. The idea of speculative tasks is that the same task will be launched in other nodes when the task works slowly, and always employs the fastest task. We design the third experiment under the condition of eight nodes, Alg2, and data size 8192×8192. The results are shown in Table 7 and Figure 9. We can see that this strategy works well in various conditions and improves the computing efficiency by 20%.

In general, the three aforementioned strategies, namely combine processing, data split optimization and the speculative task scheduling, are all employed to resolve the time-consuming data transfer issue among different nodes. The origin of this problem is the poor network performance, which puts a burden on processing, partitioning and scheduling. Therefore, we conduct another experiment with a 10 Gbps network to test the effectiveness of the three strategies. Firstly, the effect of combine processing and speculative scheduling in 10 Gbps network are relatively limited in that the difference between use and no-use is negligible. Secondly, the optimized data split number in a 10 Gbps network is nine compared with 32 in a 100 Mbps network. Finally, we can see that different strategies should be applied according to different network performances. For low speed networks, the three proposed strategies are preferred to reduce the impact of data transfer. As for high speed networks, the MapReduce model is more simple without considering optimization approaches and with no need for small data splits.

To sum up, cloud computing is an effective method to boost the SAR raw data simulation in a large scale computing network. Although the results are barely satisfactory in our mini scale cloud platform, it demonstrates the feasibility of the cloud algorithm. Its parallel efficiency is similar to the results of the other group. Moreover, two kinds of optimization strategies are introduced and can be applied to other cloud applications.

## 5. Conclusions

We have investigated the cloud computing based SAR raw data simulation algorithm. The MapReduce model is introduced to calculate the irregular signal accumulation. Meanwhile, three kinds of optimization strategies are put forward from the aspects of programming model, HDFS configuration and scheduling. The simulation experiments confirm the merits of the cloud algorithm. With the increase of cloud nodes, the simulation time is gradually reduced. The computing efficiency can be further improved by the three aforementioned strategies. Under the condition of eight cloud nodes, the method achieves a speedup about 4× over the baseline serial approach. In spite of this, higher speedup can be expected with the improvement of cloud scale. Therefore, cloud computing can fully exploit the large scale computing power, and can be used for other SAR processing related algorithms. However, the calculation of map tasks are still running on CPUs. For extensive application, we plan in the near future to introduce the GPU to strengthen the computing efficiency of cloud computing, thus realizing the fusion of HPC and cloud computing.

## Figures and Tables

**Table 1:** Experiment configuration.

Name	Configuration
operation system	Linux RedHat 5.3
Hadoop	Version 2.5.2
Java	Version 1.7
NameNode	Hex-core 3.2 GHz Intel Xeon processor, 16 GB memory
DataNode	Bi-core 3.2 GHz Intel I5 processor, 4 GB memory
Network	100 Mbps network

**Table 2:** Experimental algorithms.

Name	Methods	Map Job	Combine Job	Reduce Job
Alg1	Serialcomputing	−	−	−
Alg2	Cloud computing	$s_{a}\left(t,\tau \right)$	accumulation	accumulation
Alg3	Cloud computing	$s_{a}\left(t,\tau \right)$	−	accumulation

**Table 3:** Run time and speedup comparison with different algorithms.

Nodes	Alg1	Alg2	Alg3	Alg2	Alg3
	Run Time (s)	Run Time (s)	Run Time (s)	Speedup	Speedup
1	420	338	372	1.24	1.12
2	−	183	198	2.30	2.12
4	−	133	168	3.16	2.50
6	−	112	131	3.75	3.21
8	−	109	125	3.86	3.36

**Table 4:** Run time comparison with distributed computing method [15] under big input file conditions.

Nodes	Alg2 Run Time (s)	DC Run Time (s)
1	1320	5482
4	411	1432
8	289	784

**Table 5:** Parallel efficiency comparison between Alg2 and Alg3.

Nodes	Alg2	Alg3
	Parallel Efficiency	Parallel Efficiency
1	100%	100%
2	92%	93%
4	64%	55%
6	50%	47%
8	39%	37%

**Table 6:** The impact of split number on algorithm performance.

Splits (Map) Number	Alg2 Run Time (s)
6	253
9	193
18	151
36	122
72	176
144	251

**Table 7:** Experiment configuration.

Split Number	Speculative Switch	Alg2 Run Time (s)
36	On	172
36	Off	217
72	On	240
72	Off	254
